# Oxygen debt repayment in the early phase of veno-arterial extracorporeal membrane oxygenation: a cluster analysis

**DOI:** 10.1186/s12872-022-02794-4

**Published:** 2022-08-08

**Authors:** E. R. Kurniawati, S. Teerenstra, N. P. A. Vranken, A. S. Sharma, J. G. Maessen, P. W. Weerwind

**Affiliations:** 1 Department of Cardiothoracic Surgery, Maastricht University Medical Center+, P. Debyelaan 25, PO Box 5800, 6202 AZ Maastricht, The Netherlands; 2 Department for Health Evidence, Section Biostatistics, Radboud University Nijmegen Medical Center, Nijmegen, The Netherlands; 3 Department of Cardiology, Zuyderland Medical Center, Heerlen, The Netherlands; 4 INA Learning Labs, Bangalore, Karnataka India; 5 Cardiovascular Research Institute Maastricht (CARIM), Maastricht University, Maastricht, The Netherlands

**Keywords:** Veno-arterial extracorporeal membrane oxygenation, Adult, Oxygen debt repayment, Cluster analysis, Cardiogenic shock

## Abstract

**Introduction:**

Early oxygen debt repayment is predictive of successful weaning from veno-arterial extracorporeal membrane oxygenation (V-A ECMO). However, studies are limited by the patient cohort’s heterogeneity. This study aimed to understand the early state of oxygen debt repayment and its association with end-organ failure and 30-day survival using cluster analysis.

**Methods:**

A retrospective, single-center study was conducted on 153V-A ECMO patients. Patients were clustered using a two-step cluster analysis based on oxygen debt and its repayment during the first 24 h of ECMO. Primary outcomes were end-organ failure and 30-day survival.

**Results:**

The overall mortality was 69.3%. For cluster analysis, 137 patients were included, due to an incomplete data set. The mortality rate in this subset was 67.9%. Three clusters were generated, representing increasing levels of total oxygen debt from cluster 1 to cluster 3. Thirty-day survival between clusters was significantly different (cluster 1: 46.9%, cluster 2: 23.4%, and cluster 3: 4.8%, *p* = 0.001). Patients in cluster 3 showed less decrement in liver enzymes, creatinine, and urea blood levels. There were significant differences in the baseline oxygen debt and the need for continuous veno-venous hemofiltration (CVVH) between survivors and non-survivors (*p* < 0.05). Forty-seven patients (34.3%) migrated between clusters within the first 24 h of support. Among these patients, 43.4% required CVVH. Notably, patients requiring CVVH and who migrated to a cluster with a higher oxygen debt repayment showed better survival rates compared to those who migrated to a cluster with a lower oxygen debt repayment.

**Conclusions:**

Oxygen debt repayment during the first 24 h of V-A ECMO shows to correspond with survival, where the baseline oxygen debt value and the necessity for continuous kidney replacement therapy appear to be influential.

## Introduction

Veno-arterial extracorporeal membrane oxygenation (V-A ECMO) is a salvage therapy used in highly selected patients with severe cardiac dysfunction refractory to conventional management [1]. Despite technological advances, the mortality of V-A ECMO patients remains high (58%) [2]. The outcome of patients who undergo V-A ECMO is influenced by multiple factors, including a wide variety of etiologies, the severity of the illness, and potential complications related to ECMO [3].

Another important factor in the clinical outcome of patients undergoing V-A ECMO is oxygen debt repayment, which can be defined as the extra oxygen that must be used in the oxidative energy process after a period of hypoxia to reconvert lactic acid to glucose and decomposed adenosine triphosphate as well as creatine phosphate to their original states. Oxygen debt is directly correlated with the level of metabolic acidosis and lactic academia [4–6]. Accumulated oxygen debt without timely repayment ultimately leads to multiple organ failures and contributes to mortality [7]. Moreover, several studies reveal that lactate clearance within the initial 24 h is the most accurate predictor of outcome following critical injury [8–10]. Hence, the current study will focus on a 24-h observation period to partially eliminate confounding clinical scenarios such as complications that could influence patient outcomes [11–13].

In the cardiogenic shock setting, oxygen debt is a complex and dynamic concept. Previous studies describe the role of oxygen debt repayment through lactate clearance as a predictive factor of V-A ECMO outcome [14, 15]. Some studies suggest that serial measurement and lactate dynamic behavior are more reliable predictors for successful weaning [5, 16, 17]. However, the state of oxygen debt repayment is rarely delineated. Therefore, this study aims to investigate the state of oxygen debt repayment and its association with end-organ failure and 30-day survival by clustering patients based on the oxygen debt and its repayment.

## Methods

### Study population and design

This retrospective study was performed at a tertiary referral university hospital and included consecutive adult patients receiving V-A ECMO. Patients with pre-existing chronic kidney insufficiency (e.g., serum creatinine ≥ 170 μmol/L with or without receiving kidney replacement therapy) were excluded from the analysis.

### Study variables and endpoints

Clinical and laboratory data were collected at the time of treatment during the support using the Extracorporeal Life Support Organization (ELSO) registry forms and the patient data management system. Additional information was extracted by reviewing hospital records. The following clinical data were anonymously evaluated at six time-points (before ECMO initiation, at 2, 8, 14, 20, and 26 h of ECMO): ECMO flow, mean arterial pressure, catecholamine requirements (adrenaline, noradrenaline, dobutamine), non-catecholamine inotrope requirement (levosimendan), phosphodiesterase inhibitor (milrinone), and laboratory assessments (serum lactate, blood gasses, aspartate aminotransferase—AST, alanine aminotransferase—ALT, lactate dehydrogenase—LDH, bilirubin, creatinine, and urea).

Oxygen delivery was calculated using the following equation (Eq. 1):

1 $$DO_{2} i = Q* \frac{{\left( {Hb * 1.36 * SaO_{2} + 0.003 * PaO_{2} } \right)}}{BSA}*10$$

where DO_2_i: indexed oxygen delivery (L/min/m^2^), Q: pump rate flow (L/min), Hb: hemoglobin (g/dl), SaO_2_: arterial oxygen saturation (%), PaO_2_: partial pressure of oxygen in arterial blood (mmHg), BSA: body surface area (m^2^)

Oxygen debt was computed from lactate concentration using the following linear regression equation (Eq. 2) [18].

2 $$Oxygen\;debt\;\left( {\text{mL/kg}} \right) = - 25.26 + 13.06 \, \times \left[ {{\text{lactate}}} \right]\left( {\text{mmol/L}} \right)^{*}$$

where *Oxygen debt is only calculated when serum lactate ≥2.0 mmol/L

Total oxygen debt results from the multiplication of body weight and oxygen debt as computed by Eq. 2.

The primary outcomes of the study were end-organ failure, specifically kidney failure reflected in the necessity of continuous veno-venous hemofiltration (CVVH) treatment, and 30-day survival.

### Statistical analysis

Socio-demographic and clinical characteristics were analyzed using descriptive statistics. Categorical variables were reported as percentages and analyzed using the Chi-square or Fisher exact test, as appropriate. Continuous variables were expressed as mean ± standard deviation (SD) or median and interquartile ranges (IQR) and compared across groups using the Student unpaired t-test or the equivalent non-parametric test if applicable. Comparison between three or more groups was performed using Chi-square tests and analysis of variance (ANOVA) tests.

Cluster analysis is an exploratory analysis to identify homogenous groups of cases if the grouping is not previously known. Due to its exploratory nature, it does not make any distinction between dependent and independent variables. This method has been described elsewhere [19]. In short, the heterogeneity factors regarding patients’ characteristics, such as the etiology of the underlying disease, the severity of the illness, and patients’ responses to the treatment can be minimized by performing cluster analysis [20]. Furthermore, the pathophysiology of cardiogenic shock is complex and not fully understood [21]. Hence, cluster analysis may help to identify homogenous subgroups within data. Due to its nature as an unsupervised method, cluster analysis was more appropriate than supervised methods such as regression or classification. The unsupervised method means that the output is unknown and there is no assumption made about the possible associations within the data [22]. K-means cluster was used due to its fast, robust and efficient nature that gives reliable results by using centroids to cluster each data point into a certain cluster based on how close the features are to the centroid [23].

Subgroups of patients or clusters with similar states of oxygen debt and oxygen debt repayment dynamic in each time interval were based on z-scores of total oxygen debt at each time point, i.e., pre-ECMO, 2, 8, 14, 20, and 26 h. Cluster analysis was based on the beginning and end of each time interval to observe the changes over time in oxygen debt and patient distribution in each cluster. The latter portrayed the migration of patients between clusters over time.

The clustering procedure started with a two-step cluster analysis algorithm with automatic determination of the number of clusters based on two variables, the z-scores at baseline and 2 h of support for the initial cluster classification. The two-dimensional centers of these initial clusters were calculated as the mean z-score at baseline and the mean z-score at 2 h of these initial clusters. Next, these centers were used as initial centers for the K-means cluster analysis to obtain the final clusters and their centers between pre-ECMO and 2 h of support. Subsequently, the centers of the final clusters during 0–2 h of support served as initial centers for the K-means cluster analysis of the z-scores at 2 and 8 h of ECMO. This procedure was repeated at the subsequent time intervals for the next sequential period between 8–14, 14–20, and 20–26 h of support, respectively. The motivation to use the final clusters of the previous time interval as starting values for the next time interval was to identify the change of centers of the clusters over time.

All patients were clustered sequentially to observe different states of oxygen debt at each time point and to distinguish between groups of patients who did and did not migrate between clusters during the early phase of the treatment.

The migration of patients between clusters was analyzed at each time interval, as well as the dynamics of clinical and laboratory parameters included in the study. Analysis of variance (ANOVA) was used to examine differences among cluster means. Tests were considered statistically significant at a 95% confidence interval (*p*-value < 0.05). Statistical analyses were performed using SPSS version 25 (SPSS Inc., Chicago, IL, USA).

## Results

### General characteristics

Baseline demographics, including age, gender, BSA, ECMO indication, cannulation site, the duration of ECMO, the necessity of IABP and CVVH for the entire cohort before cluster analysis was performed are summarized in Table 1.

**Table 1:** Patients’ clinical and laboratory pre-ECMO characteristics of the study population

Variable	Group		*p*-value
	Non-survivors (n = 106)	Survivors (n = 47)	
Age (years)	62 ± 13	61 ± 12	0.564
Gender (male (%))	68 (64.2%)	33 (70.2%)	0.465
BSA (m^2^)	2 ± 0.2	1.9 ± 0.2	0.730
Indexed DO_2_ (L/min/m^2^)	267.4 ± 69	297.1 ± 60.9	0.057
ECMO indication			
Arrhythmia	1 (0.9%)	3 (6.4%)	0.019
Postcardiotomy heart failure	54 (50.9%)	29 (61.7%)	
Cardiogenic shock	30 (28.3%)	10 (21.3%)	
ECPR	17 (16.1%)	1 (2.1%)	
Others*	4 (3.8%)	4 (8.5%)	
Decision to initiate ECMO within 8 h of onset of failure (Yes (%))	81 (76.4%)	46 (97.9%)	0.001
Cannulation			
Femoro-femoral	92 (86.8%)	36 (76.6%)	0.253
Femoral-aortic	13 (12.3%)	9 (19.1%)	
Atrio-aortic	1 (0.9%)	1 (2.1%)	
Femoral-pulmonary artery	0 (0%)	1 (2.1%)	
ECMO duration (hours)	79.4 ± 93.4	150 ± 137.9	< 0.001
IABP (yes (%))	29 (27.4%)	16 (34%)	0.403
CVVH (yes (%))	44 (41.5%)	12 (25.5%)	0.058

*BSA* Body Surface Area; *DO*_2_ oxygen delivery; *ECMO* Extracorporeal Membrane Oxygenation; *ECPR* Extracorporeal Cardio Pulmonary Resuscitation, only limited to patients underwent ECPR; *IABP* Intra-Aortic Balloon Pump; *CVVH* Continuous Veno-Venous Hemofiltration

^*^Others include myocardial stunning and tamponade

The average age of the 153 patients included in this study was 62 years, with a proportion of 66% male patients (n = 101). ECMO was used as the primary device to support all the patients. The overall mortality rate in this study population was 69.3%. The median duration of ECMO was 70.5 [17.4–147.2] hours. Based on ECMO indication, the highest mortality rate (94.4%) was found in patients undergoing extracorporeal cardiopulmonary resuscitation (ECPR). Conversely, patients with cardiac arrhythmia as an indication for ECMO showed the highest survival rate (75%, *p* = 0.019). Although, this indication only accounted for four patients (2.6%) of the total cohort. The decision to start ECMO within eight hours of the onset of cardiac failure was differed significantly between non-survivors (76.4%) and survivors (97.9%) (*p* = 0.001). In the non-survivor group, ECMO was discontinued in 41.5% of the patients during the first 24 h due to an unfavorable prognosis. The ECMO duration in survivors was almost twice as long as for non-survivors (*p* < 0.001). Indexed DO_2_ was similar between survivors and non-survivors (*p* = 0.057). Among the 56 patients with acute kidney failure and treated with CVVH, merely 21.4% survived.

### Cluster analysis

**Fig. 1:**
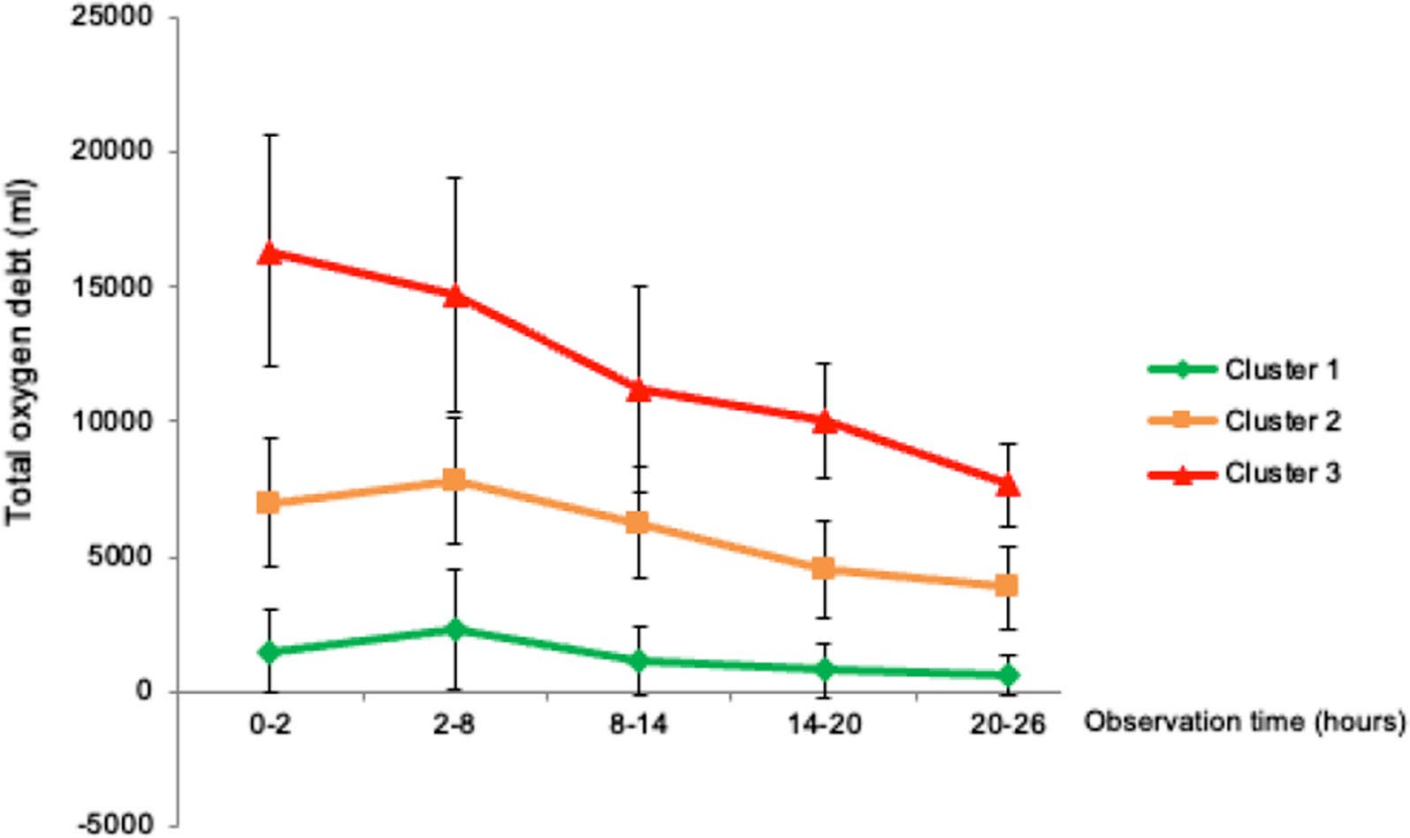
Clusters based on oxygen debt during the first 24 h of ECMO

A two-step cluster analysis was performed for the initial grouping within the patient population, generating three groups based on similar oxygen debt and its compensation (Fig. 1). The Z-scores of oxygen debt per body weight pre-ECMO and 2 h after initiating ECMO was used for initial clustering. During cluster analysis, 16 patients could not be analyzed, most likely due to incomplete data, leaving 137 patients to be incorporated in the cluster analysis with the mortality rate of 67.9%. Three clusters were generated automatically during the analysis with sufficient numbers of patients in each cluster. Results showed that the oxygen debt was the lowest in cluster 1, higher in cluster 2 and highest in cluster 3 at all-time points. Meanwhile, indexed DO_2_ was similar between clusters throughout the observation period (Table 2). There were significant differences in the baseline oxygen debt and the necessity of CVVH between survivors and non-survivors (*p* < 0.05 for both comparisons).

**Table 2:** Mean indexed DO_2_ (L/min/m^2^) across clusters in the first 24 h of support

Time point	Cluster			*p*-value
	1	2	3	
Pre-ECMO—2 h after initiation	291.8 ± 77.9	295.9 ± 89.9	263.9 ± 73.7	0.381
2–8 h after initiation	299.6 ± 75.0	278.0 ± 82.1	260.8 ± 65.8	0.159
8–14 h after initiation	287.7 ± 86.4	280.8 ± 82.3	244.9 ± 81.1	0.311
14–20 h after initiation	275.8 ± 83.5	276.6 ± 49.0	245.7 ± 121.9	0.758
20–26 h after initiation	276.5 ± 67.1	267.2 ± 50.9	282.1 ± 52.9	0.499

*DO*_2_ oxygen delivery; *ECMO* Extracorporeal Membrane Oxygenation

**Fig. 2:**
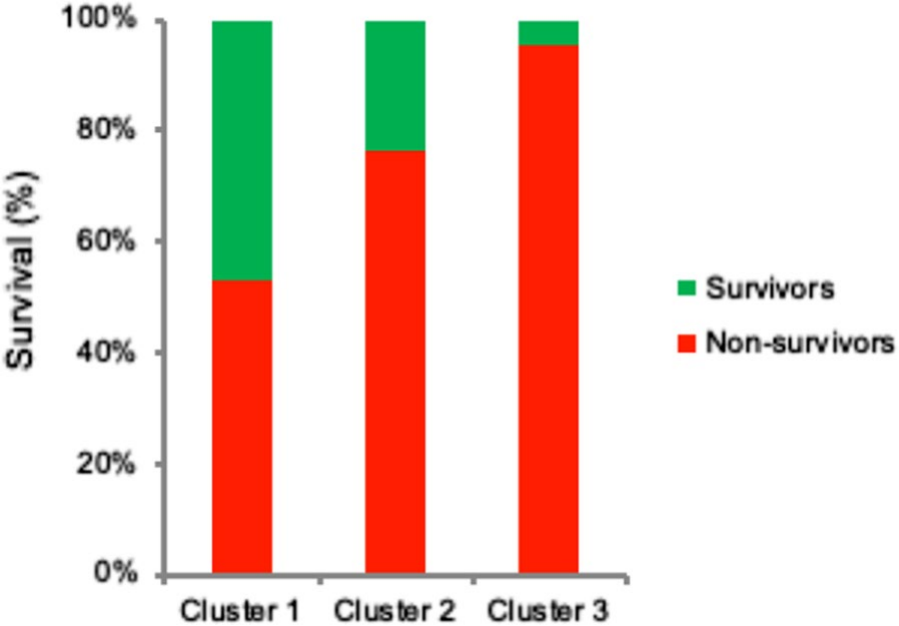
30-day survival rate per cluster

As shown in Fig. 2, there was a statistically significant difference in 30-day survival between clusters (cluster 1: 46.9%, cluster 2: 23.4%, and cluster 3: 4.8%; *p* = 0.001). The indication for ECMO was similar across clusters (*p* = 0.081), in contrast to the support duration which differed significantly between clusters with an average support time of 146 (cluster 1), 89 (cluster 2), and 34 h (cluster 3), respectively (*p* < 0.001).

Hemodynamic parameters, including mean arterial pressure and ECMO flow, as well as infusion rate of vasoactive and inotropic drugs, were compared between the clusters at all-time points. The results showed a significant difference between clusters for mean arterial pressures at 8, 14, and 20 h after ECMO initiation (*p* < 0.05). Noradrenaline (norepinephrine) dosage differed significantly across all-time points except before ECMO initiation (*p* = 0.101). A trend toward a higher noradrenaline requirement was observed among the patients designated to clusters 2 and 3. It implies that patients in cluster 1 had the lowest noradrenaline requirements, in contrast to the patients in cluster 3, who showed to have received the highest dosage of noradrenaline. No difference in ECMO flow, the dosage of other vasoactive and inotropic drugs was observed between the clusters at all time points (Appendix).

Metabolic parameters such as pH, base excess, and lactate concentration differed significantly between clusters at all time points. Patients in a cluster with a better survival rate showed a trend towards a higher pH and a lower base excess and serum lactate concentration. A similar trend was observed in the dynamics of liver and kidney function parameters (Appendix). Liver enzymes (AST and ALT) were significantly different between clusters at all time points (*p* < 0.05), except at pre-ECMO (*p* > 0.05). LDH levels before ECMO initiation until 20 h of ECMO differed significantly between clusters (*p* < 0.05). Bilirubin levels were similar between clusters throughout the observational period, except for 2 h after initiation of support. Creatinine and urea, as kidney function parameters, differed at several time points during the observational period. Urea showed to be significantly different at 2 and 20 h after ECMO initiation, while creatinine showed differences before ECMO initiation and at 8 h of support (Appendix).

In total, 65.7% did not migrate during the observational period. The majority of these patients (N = 62) did not survive (cluster 1 N = 35, cluster 2 N = 11, cluster 3 N = 16). The median age within this group of patients was lower in survivors (61 [55–69] years) compared to non-survivors (65 [51–70] years). Meanwhile, the survivors had significantly lower median baseline oxygen debt compared to non-survivors in non-migrated patients (3.0 [2.0–4.6] vs 7.5 [3.0–14.5] mmol/L, *p* < 0.001). The majority of the non-migrated patient group (67.8%) did not survive, including all patients who initially belonged to clusters 2 and 3, and 34 of 62 (54%) patients in cluster 1 (Table 3).

**Table 3:** Patients clusterization and migration during the first 26 h of ECMO

Cluster	Non-survivors (n = 94)	Survivors (n = 43)	Total
1 (no migration), n(%)	34 (54%)	29 (46%)	63
1 migrated to 2, n(%)	8 (72%)	3 (27%)	11
1 migrated to 3, n(%)	4 (80%)	1 (20%)	5
2 (no migration), n(%)	11 (100%)	0 (0%)	11
2 migrated to 1, n(%)	11 (52%)	10 (48%)	21
2 migrated to 3, n(%)	5 (100%)	0 (0%)	5
3 (no migration), n(%)	16 (100%)	0 (0%)	16
3 migrated to 1, n(%)	1 (50%)	1 (50%)	2
3 migrated to 2, n(%)	3 (100%)	0 (0%)	3

Conversely, 34.3% migrated between clusters during the observational time. Most of these patients (N = 32) that showed inter-cluster migration during the early support phase did not survive. Over half of these patients (N = 17) moved to a cluster with a worse outcome (referred to as a ‘’lower’’ cluster), and 15 patients migrated to a cluster with a more favorable outcome (referred to as an ‘’upper’’ cluster). The baseline oxygen debt differed significantly between survivors and non-survivors in those who migrated to a lower cluster (median of 2.5 [2.2–3.0] vs 3.9 [2.2–6.0] mmol/L for survivors vs non-survivors, respectively, *p* = 0.027). Among the patients who migrated to a lower cluster, the baseline of oxygen debt was significantly lower in patients treated without CVVH compared to those treated with CVVH (median of 3.1 [2.2–3.6] vs 4.4 [2.2–8.3] mmol/L, *p* = 0.008). Furthermore, the majority of the survivors migrated to an upper cluster (N = 11). In general, patients who migrated from one cluster to another and survived were relatively younger with lower baseline oxygen debt (median age 62 [53–70] years; median oxygen debt 6.5 [3.3–8.3] mmol/L), in contrast to patients that did not survive (median age 66 [56–70] years; median oxygen debt 7.6 [3.5–10.4] mmol/L). Another noticeable observation was that although four survivors migrated from an upper to a lower cluster, this particular subset of patients showed lower baseline oxygen debt averaging 2.5 mmol/L.

Meanwhile, 15 patients who migrated to an upper cluster and did not survive showed relatively high baseline oxygen debt, on average 10.4 mmol/L. Cluster migrations during the first 24 h of the support are denoted in detail in Table 3.

Among 53 patients with acute kidney failure requiring CVVH, 43.4% of the patients migrated between clusters during the first 24 h. Over half of these patients (N = 13) migrated to a lower cluster, and merely two patients survived. On the other hand, the survival rate of patients requiring CVVH and exhibiting a migration to an upper cluster doubled (N = 10). The survivors treated with CVVH who migrated between clusters during the first 24 h showed lower median oxygen debt and creatinine levels at baseline initiation (5.2 [2.2–6.3] mmol/L and 112 [87.5–154] μmol/L, respectively) and at 24 h after ECMO initiation (3.1 [2.4–7.8] mmol/L and 161 [100.3–266] μmol/L, respectively) compared to patients with CVVH and inter-cluster migration but did not survive (7.7 [4.2–10.6] mmol/L and 142 [107.5–197.5] μmol/L; 4.3 [2.0–7.4] mmol/L and 235.5 [177–260.3] μmol/L, respectively).

## Discussion

This observational study focused on the early dynamics of oxygen debt repayment in relation to the development of end-organ failure and 30-day survival of patients treated by V-A ECMO. Using cluster analysis, the heterogeneous cohort was grouped into three homogeneous clusters based on the level of oxygen debt and its repayment rate. Clinical outcome was ameliorated in patients exhibiting an initial low oxygen debt or a faster repayment rate, in contrast to patients with a higher initial oxygen debt or a delayed oxygen debt repayment rate. Thus, a comprehensive understanding of the inter-cluster patient migration may help tailor the ECMO treatment to better suit the patient’s condition [20].

An important observation in this study was the movement within the clusters, where all clusters demonstrated an oxygen debt peak at 2 h of support with a subsequent decrease. The dynamics of oxygen debt repayment were observed during the first 24 h after ECMO initiation, and statistically significant associations with clinical outcome parameters were found. During the first 24 h of support, patients with adequate oxygen debt compensation migrated to a cluster with a higher survival rate and patients with inadequate oxygen debt compensation migrated to a cluster with a lower survival rate. Hence, patients initially belonging to clusters 2 and 3 and did not show any migration during the early phase of support did not survive. Similar findings are described by Cheng-Long et al., where early lactate behavior showed to be predictive of clinical outcome in 123 adult V-A ECMO patients following postcardiotomy cardiogenic shock [16]. Moreover, those who migrated and survived had lower mean lactate levels at baseline compared to those who migrated and did not survive. Earlier studies reported that pre-ECMO blood lactate is independently associated with increased mortality risk in post-cardiogenic shock patients treated with V-A ECMO [24, 25].

In the current study, the average V-A ECMO duration of patients in cluster 1 was longer (6.1 days) than in patients in cluster 2 (3.7 days) and cluster 3 (1.4 days). This is in line with the results by Smith et al., in which the authors stated that an ECMO duration of fewer than four days is associated with a decreased chance of survival as mortality is high, especially in the first few days of V-A ECMO [26]. Common reasons for early weaning are organ failure, a confirmed unfavorable prognosis, or as per request of the patient’s next of kin for termination of support [26]. The fact that survivors are generally supported for a more extended period than non-survivors can be interpreted as a manifestation of ECMO’s role as a bridge to recovery. However, mortality increases after one week of V-A ECMO initiation, which is associated with the underlying disease progression or the ECMO complications that supervene and preclude the chance of survival [26].

Another factor contributing to outcome parameters, including survival, is the time span between the onset of cardiac failure and the decision to initiate V-A ECMO. Early initiation of the treatment contributes to improved ECMO outcome [27, 28], while delayed initiation of ECMO is associated with higher mortality rates [29, 30]. This can be attributed to a prolonged period of suboptimal tissue perfusion, resulting in unfavorable consequences in terms of end-organ function [31]. This is supported by the current results showing that in nearly all surviving patients (97.8%), ECMO treatment was initiated at a relatively early stage, i.e., within eight hours after the onset of cardiac failure. These results are in accordance with previous studies suggesting that early ECMO initiation is essential in contributing toward more favorable clinical and neurological outcomes [32, 33]. Conversely, premature initiation of ECMO may lead to an increase in hospital costs while patients are potentially unnecessarily exposed to complications associated with ECMO [30]. Therefore, a thorough assessment of indication followed by timely ECMO initiation and weaning are essential during the treatment, leading to more favorable outcomes.

Regardless of significant differences in initial oxygen debt between clusters in the current cohort, support flows were relatively similar. One might speculate that if the patients with higher initial total oxygen debt were supported by higher pump flows, oxygen debt repayment would commence at a higher pace. However, there is a paradox during cannula size selection due to cannula restrictions where anatomical considerations and the desired targeted flow rate need to be taken into account, which in turn determines the maximum pump flow rate [34]. Moreover, using larger cannulas in patients with a higher oxygen debt might introduce an increased risk of vascular complications as well as lower limb ischemia [35, 36].

During the support, arterial blood pressure is one of the most fundamental factors to be taken into account when managing V-A ECMO [37], since hypotension is related to end-organ hypoperfusion and a decline in kidney function [38], often linked to mortality [39]. Sufficient mean arterial pressure is necessary for adequate end-organ perfusion, especially for organ systems, including the brain and kidneys [37]. Previous studies affirmed that maintaining a mean arterial pressure above 65 mmHg is generally adequate [40, 41], and that mean arterial pressures between 70 and 80 mmHg are more likely to result in better outcomes [37]. Furthermore, signs of improving cardiac function during V-A ECMO include increasing blood pressures, increasing pulsatility of the arterial pressure waveform, and reduced need for inotropic and vasopressors [42]. The latter was confirmed in the current study, as patients who migrated to clusters 1 and 2 showed higher mean arterial pressures (70–80 mmHg) compared to patients in cluster 3 (< 65 mmHg). Additionally, patients who migrated to a lower cluster showed subclinical creatinine and urea levels, indicating kidney insufficiency. Based on the cluster analysis, patients who migrated to an upper cluster required noradrenaline less frequently and vice versa. As a vasopressor, noradrenaline helps to target the mean arterial pressure in patients with hypotension (< 60 mmHg) [43]. Hence, patients in cluster 3 generally required the highest noradrenaline doses.

V-A ECMO provides temporary circulatory support to allow recovery of end-organ function in patients with severe hemodynamic instability associated with multi-organ failure [44]. Multi-organ failure such as liver and kidney failure [45] is a common cause of mortality during V-A ECMO management [46–48]. In the current study, we observed that 36.6% of the V-A ECMO patients required kidney replacement therapy, which is more frequent compared to the results reported by the ELSO registry (12%) [49]. Further, the current survival rate of 21.4% among V-A ECMO patients receiving CVVH is lower than the 36.1% 30-day survival rate among V-A ECMO without CVVH. Dialysis therapy following acute kidney injury is an independent risk factor for mortality for ECMO patients [38]. On the other hand, Chang et al. described similar survival rates for ECMO patients who did and did not receive CVVH (25% vs 35%) [50]. Notably, in the present study, 48.9% of the V-A ECMO patients who migrated between clusters during the observation time received CVVH. In this cohort, the survival rate for V-A ECMO patients receiving CVVH was similar whether they did or did not migrate between clusters (21.7% vs 21.4%, respectively). Nonetheless, V-A ECMO patients receiving CVVH who were able to repay their oxygen debt in a timely manner seemed to have better clinical outcomes. Current findings illustrate that the survival rate of the patients who migrated to a cluster with better oxygen debt repayment was almost twice as high (30.0%) compared to those who migrated to a cluster with a lower oxygen debt repayment rate (15.4%).

Enzymes including AST, ALT, LDH, and the product of hemoglobin catabolism, bilirubin, are the common markers of liver damage in critically ill patients [51, 52], of which AST and ALT are considered the most specific biomarkers for liver damage [53]. Elevated levels of these enzymes are associated with prolonged stay in the intensive care unit and early mortality regardless of adequate cardiopulmonary support [54, 55]. Current results showed that ALT levels were more than five times the normal upper limit range, indicating severe liver damage in this patient population [56]. A trend of elevated AST and ALT levels from cluster 1 through cluster 3 was observed, indicating a more severely impaired liver function in patients in cluster 3 than in clusters 1 and 2. Moreover, current bilirubin results did not significantly differ between the three clusters, while Roth et al. indicated total bilirubin as one of the sensitive parameters for predicting the short-term and long-term outcomes in V-A ECMO patients [55]. Generally, ECMO therapy may affect liver perfusion due to severe hemodynamic fluctuations in pump flow and the lack of pulsatility, rapid changes of vasopressors doses after ECMO initiation, and air or thrombus embolization [57, 58]. Roth et al. argued that this hemodynamic instability led to the pronounced impact of bilirubin and alkaline phosphatase on clinical outcomes in their patients supported by V-A ECMO [55]. These findings merit a comprehensive study investigating the impact of the hepatic biochemical profile on clinical outcomes in V-A ECMO patients.

Several limitations in the current study need to be considered. First, this was a retrospective single-center study. Thus, several limitations inherent to the nature of the retrospective study design should be acknowledged, including partially missing data and ascertainment errors. Therefore, prospective multicenter studies are warranted with a specific focus on optimal treatment modalities of the different patient subsets. Secondly, the relatively small population may have affected the statistical power and thereby the ability to identify potential differences between subsets. Hence, further studies with a larger patient population are required.

## Conclusions

Oxygen debt repayment during the first 24 h of V-A ECMO shows to correspond with survival, where the baseline oxygen debt value and the prerequisite for continuous kidney replacement therapy appear to be influential factors.

## Figures and Tables

**Fig. 3:**
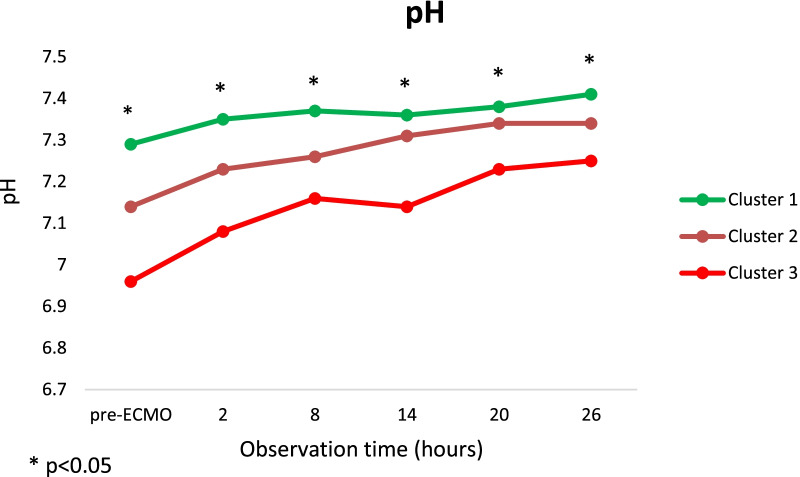
Blood pH per cluster during the first 26 h of V-A ECMO

**Fig. 4:**
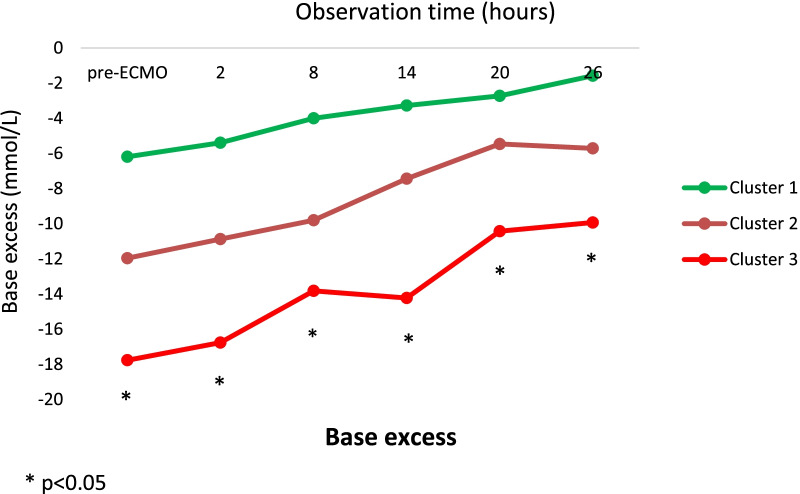
Base excess per cluster during the first 26 h of V-A ECMO

**Fig. 5:**
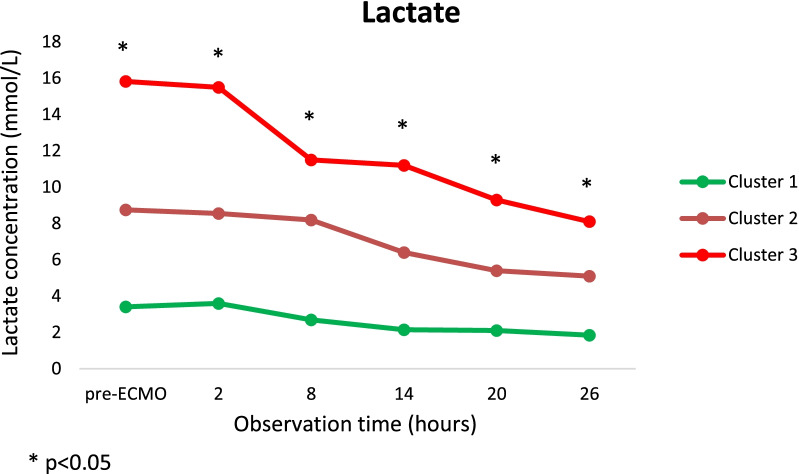
Lactate level per cluster during the first 26 h of V-A ECMO

**Fig. 6:**
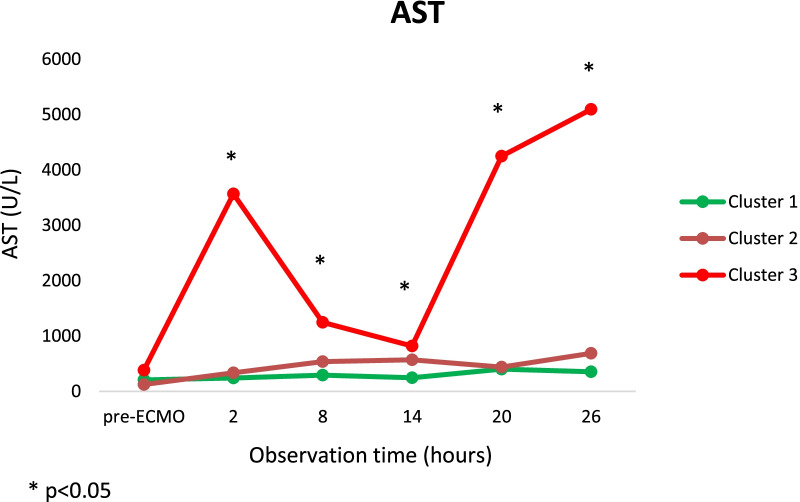
AST per cluster during the first 26 h of V-A ECMO

**Fig. 7:**
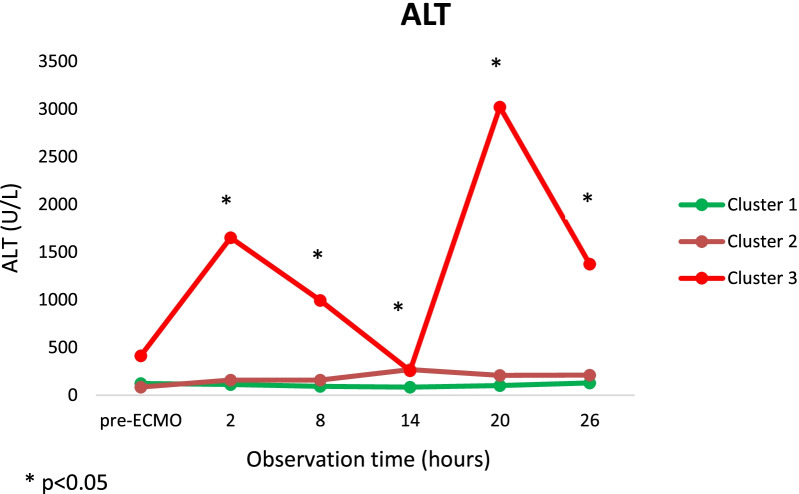
ALT per cluster during the first 26 h of V-A ECMO

**Fig. 8:**
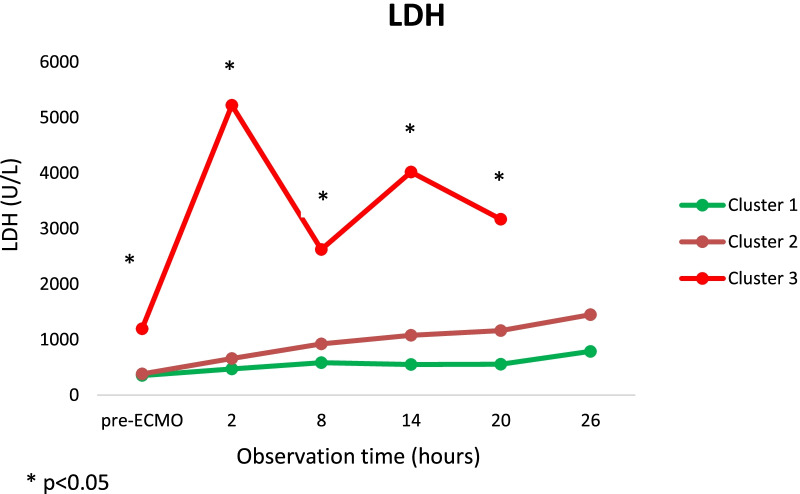
LDH per cluster during the first 26 h of V-A ECMO

**Fig. 9:**
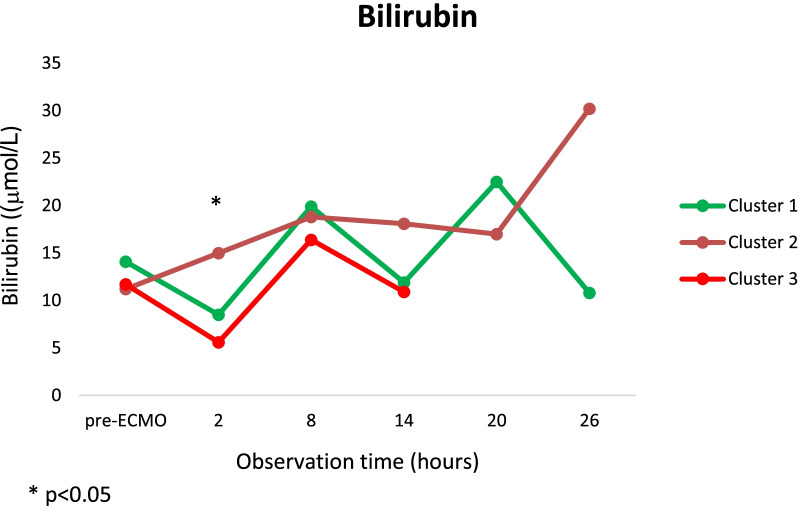
Bilirubin level per cluster during the first 26 h of V-A ECMO

**Fig. 10:**
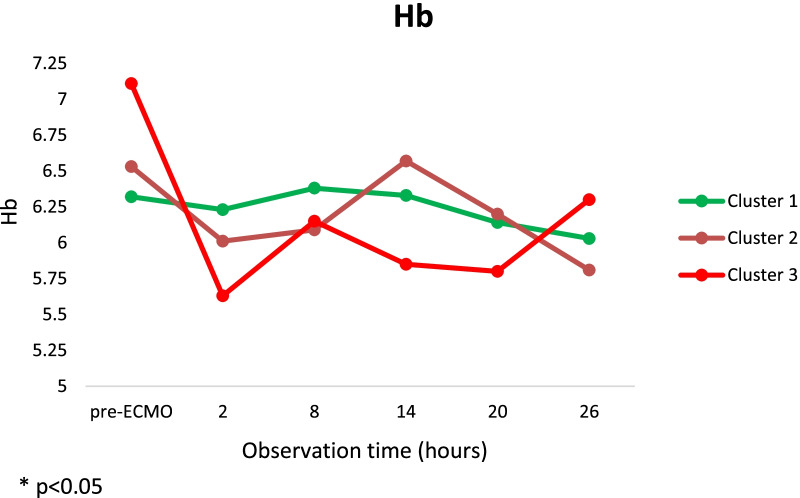
Hemoglobin level per cluster during the first 26 h of V-A ECMO

**Fig. 11:**
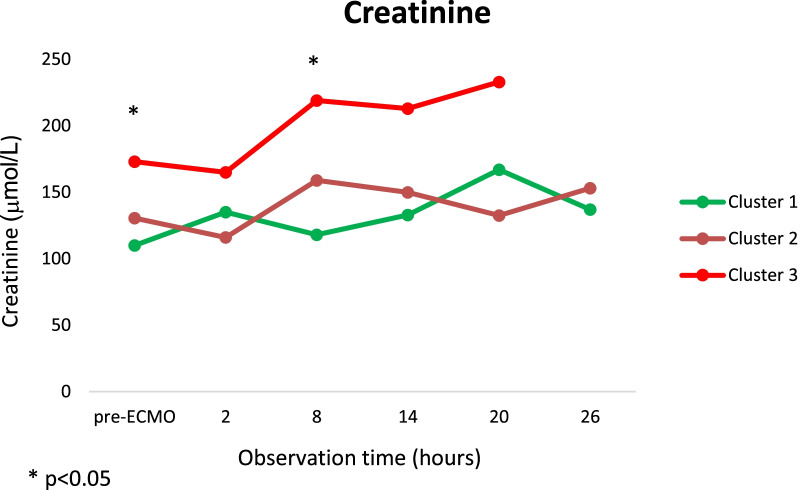
Creatinine level per cluster during the first 26 h of V-A ECMO

**Fig. 12:**
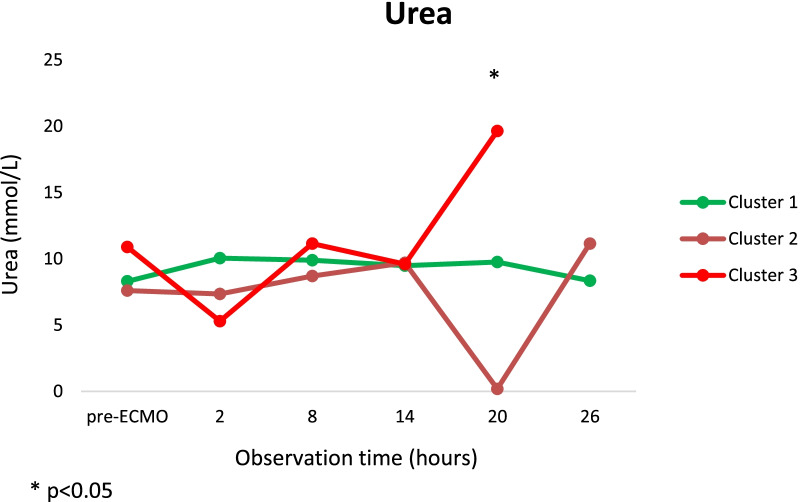
Urea level per cluster during the first 26 h of V-A ECMO

**Fig. 13:**
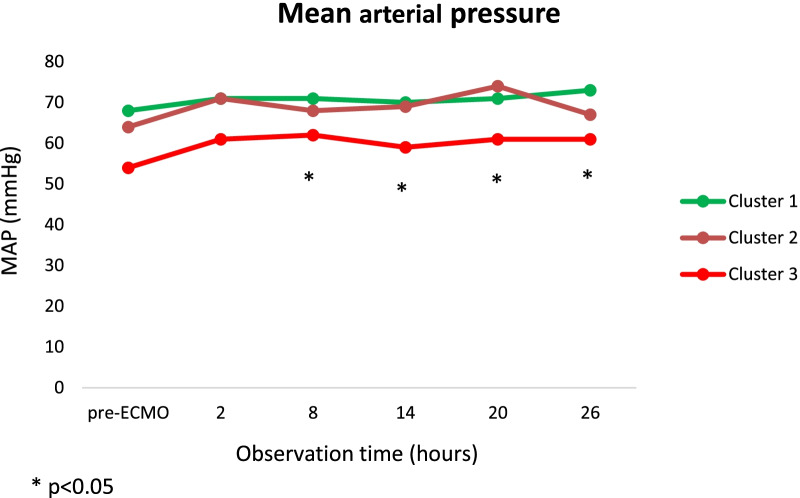
Mean arterial pressure per cluster during the first 26 h of V-A ECMO

**Fig. 14:**
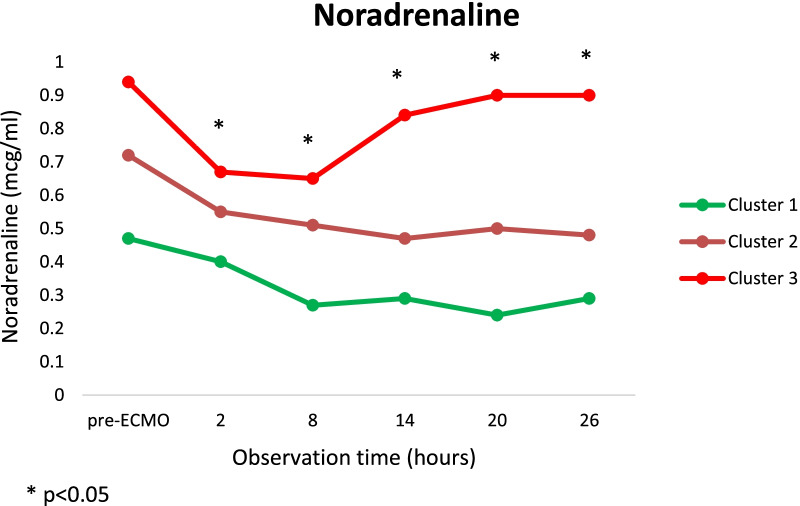
Noradrenaline dosage per cluster during the first 26 h of V-A ECMO

**Fig. 15:**
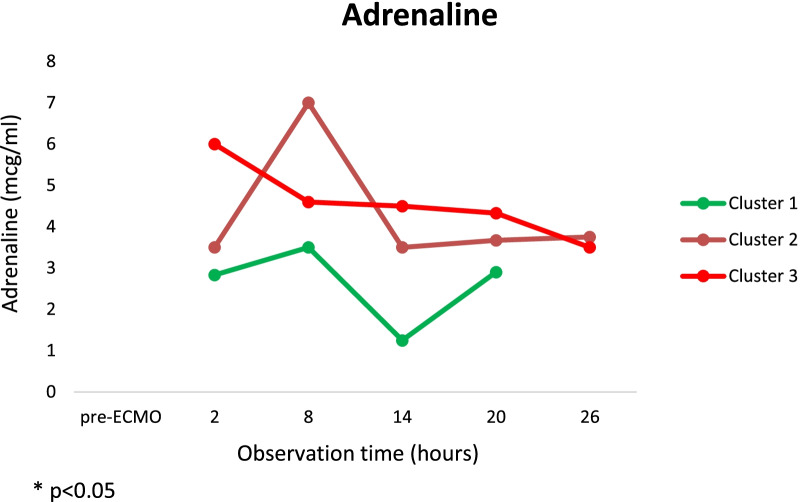
Adrenaline dosage per cluster during the first 26 h of V-A ECMO

**Fig. 16:**
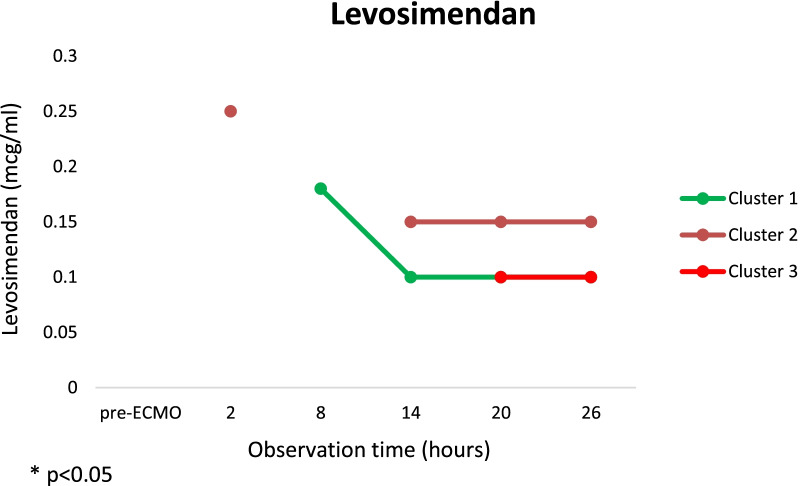
Levosimendan dosage per cluster during the first 26 h of V-A ECMO

**Fig. 17:**
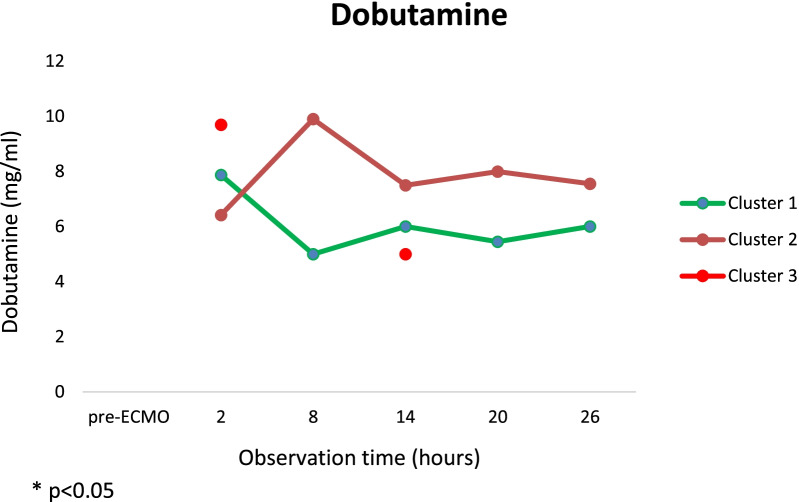
Dobutamine dosage per cluster during the first 26 h of V-A ECMO

**Fig. 18:**
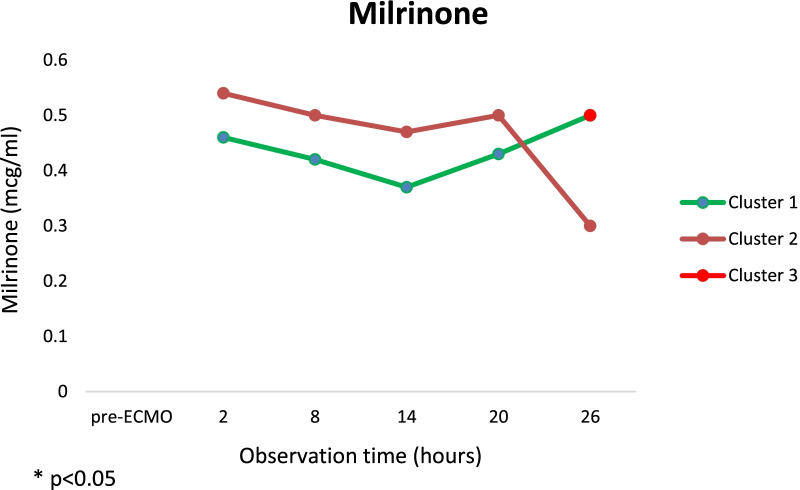
Milrinone dosage per cluster during the first 26 h of V-A ECMO

## Data Availability

The datasets generated during and analysed during the current study are not publicly available due to limitation of ethical approval involving the patient data and anonymity but are available from the corresponding author on reasonable request.

## Appendix

See Figs. 3, 4, 5, 6, 7, 8, 9, 10, 11, 12, 13, 14, 15, 16, 17 and 18.
